# Gut microbiota composition in health-care facility-and community-onset diarrheic patients with *Clostridioides difficile* infection

**DOI:** 10.1038/s41598-021-90380-7

**Published:** 2021-05-25

**Authors:** Giovanny Herrera, Laura Vega, Manuel Alfonso Patarroyo, Juan David Ramírez, Marina Muñoz

**Affiliations:** 1 Centro de Investigaciones en Microbiología y Biotecnología – UR (CIMBIUR), Facultad de Ciencias Naturales, Universidad del Rosario, Bogotá, Colombia; 2 Molecular Biology and Immunology Department, Fundación Instituto de Inmunología de Colombia (FIDIC), Bogotá, Colombia; 3 Microbiology Department, Faculty of Medicine, Universidad Nacional de Colombia, 111321 Bogotá D.C., Colombia; 4 Health Sciences Division, Main Campus, Universidad Santo Tomás, 110231 Bogotá D.C., Colombia

**Keywords:** Bacteria, Microbial communities, Pathogens

## Abstract

The role of gut microbiota in the establishment and development of *Clostridioides difficile* infection (CDI) has been widely discussed. Studies showed the impact of CDI on bacterial communities and the importance of some genera and species in recovering from and preventing infection. However, most studies have overlooked important components of the intestinal ecosystem, such as eukaryotes and archaea. We investigated the bacterial, archaea, and eukaryotic intestinal microbiota of patients with health-care-facility- or community-onset (HCFO and CO, respectively) diarrhea who were positive or negative for CDI. The CDI-positive groups (CO/+, HCFO/+) showed an increase in microorganisms belonging to Bacteroidetes, Firmicutes, Proteobacteria, Ascomycota, and Opalinata compared with the CDI-negative groups (CO/−, HCFO/−). Patients with intrahospital-acquired diarrhea (HCFO/+, HCFO/−) showed a marked decrease in bacteria beneficial to the intestine, and there was evidence of increased Archaea and *Candida* and *Malassezia* species compared with the CO groups (CO/+, CO/−). Characteristic microbiota biomarkers were established for each group. Finally, correlations between bacteria and eukaryotes indicated interactions among the different kingdoms making up the intestinal ecosystem. We showed the impact of CDI on microbiota and how it varies with where the infection is acquired, being intrahospital-acquired diarrhea one of the most influential factors in the modulation of bacterial, archaea, and eukaryotic populations. We also highlight interactions between the different kingdoms of the intestinal ecosystem, which need to be evaluated to improve our understanding of CDI pathophysiology.

## Introduction

Health-care-associated infections (HCAIs) are a high-impact issue worldwide, as they favor the development of diseases that put the patient’s life at risk and are associated with high expenditure rates within health systems^1^. One HCAI with the greatest global impact is *Clostridioides difficile* Infection (CDI), considered to be the causative agent of diarrhea associated with the use of antibiotics^2–4^. This microorganism can cause a range of problems from asymptomatic infections, dehydration, and diarrhea to severe digestive tract complications, such as toxic megacolon, pseudomembranous colitis, and sepsis, and even death^2,3,5^. The problems associated with CDI have worsened as a result of increased incidence and mortality, mainly in patients of the intensive care unit (ICU), where it is reported as being one of the five infections with the greatest impact worldwide^4,5^.

Various studies have shown that the influence of CDI on the intestinal microbiota is characterized by a disruption and alteration of its homeostasis, leading to various consequences including diarrhea^6–9^. Despite the absence of a definition for a core microbiome among individuals with CDI, because of the interindividual variability that may exist, clear differences have been found between groups of people with positive or negative CDI status. Among the main alterations suffered by people with CDI is a decrease in particular bacterial populations, such as *Bacteroides*, *Lachnospiraceae*, and *Ruminococcaceae*, which is accompanied on many occasions by an increase in bacteria of the phylum Proteobacteria, as well as a decrease in microbiota bacterial diversity^9–11^*.* Similarly, various groups of bacteria with the ability to inhibit the growth of *C. difficile*, both in vivo and in vitro, have been described as being associated with CDI, including *C. scindens*, *B. adolescents,* and some members of the *Lachnospiraceae* family^9,11,12^.

The role of eukaryotes in the development of CDI has been addressed in only a few investigations in which the involvement of fungi was emphasized^13,14^. Among the main findings was an increase in the relative abundance of fungi of the genus *Penicillium* in patients with CDI compared with patients without CDI^15^, as well as a relationship between *C. albicans* and failures in fecal transplant treatment^16^. Recent studies have shown the co-occurrence of *C. difficile* and *Blastocystis*^14^. However, the impact of this eukaryote on intestinal microbiota in relation to CDI has not been clarified.

A description of the composition and abundance of bacterial species, both in healthy individuals and those suffering from disorders related to CDI, has promoted the understanding of various aspects of the pathophysiology of this disease^9,17^. However, the roles played by other organisms that are part of the microbiota, such as archaea and eukaryotes, has not been addressed in recent studies. Therefore, the impacts of both the gut microbiota and the established interactions between the different members of the microbiota remain poorly understood^18^. Furthermore, most of these studies have been conducted in Europe and North America, and we are lacking a description of the microbiota of patients with CDI in South America and, particularly, Colombia. Therefore, this study proposed to determine the intestinal microbiome (including bacteria, archaea, and eukarya) of patients with diarrhea acquired at the intrahospital or community level under either positive or negative CDI statuses. A marked decrease in the relative abundance of bacteria, such as *Dorea*, *Faecalibacterium*, *Lachnospira*, and *Prevotella*, was evidenced in the groups positive for CDI, and there was an increase in fungi of the genus *Candida* in CDI-positive patients with diarrhea acquired in hospital. Inverse correlations were observed between some groups of bacteria and eukaryotes. Finally, the associations among bacterial and eukaryotic families and genera with CDI were investigated.

## Results

### Compositional differences between groups

The 98 samples used in this study came from diarrheal patients treated in two fourth-level hospitals in Bogotá, which serve an urban population with different clinical, sociodemographic, and economic characteristics. The diversity of specialties served by the hospital guarantees a high variability in the patients included in the study. Of the 48 patients in total included in the HCFO/− and HCFO/+ groups, data were collected for 32 of them. The HCFO/− group comprised eight women and six men aged between 18 and 81 years (${\overline{\text{x}}}$  = 66.2, SD = 20.4). In the HCFO/+ group, there were 10 women and eight men aged between 26 and 92 years (${\overline{\text{x}}}$ = 64.5, SD = 14.7). No sociodemographic data were obtained for the CO cases. When reviewing the quality of the sequencing, an average of 350,000 reads were obtained per sample, with a minimum of 200,000 and a maximum of 400,000 reads, which was adequate to determine the diversity in each of the samples considering the rarefaction analysis indicated a minimum of 60,000 reads were needed to reveal diversity (Figure S1). We also found no sequences with ambiguous assignment in any position, and more than 99.9% of the reads had a phred score of more than 30, thus we decided that no sequences needed to be removed before analysis.

**Figure 1:**
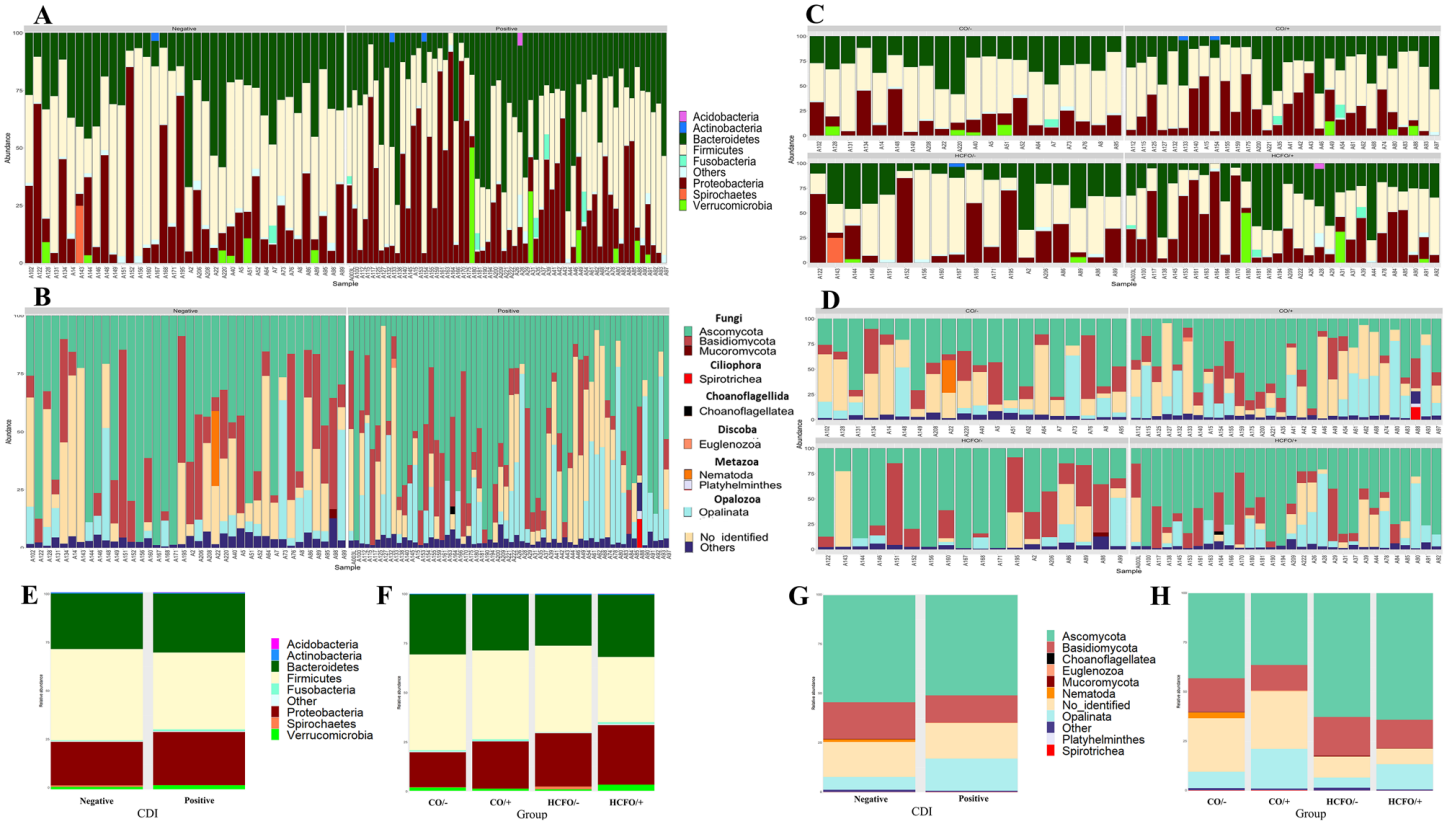
Microbial composition of diarrheic patients’ gut microbiota by CDI status and by group (HCFO/+ , HCFO/−, CO/+, CO/−). (**A**) Bar plots showing the 9 major bacterial phyla by CDI status. (**B**) Bar plots of major eukaryotic groups by CDI status. (**C**) Bar plots of major bacterial phyla by group. (**D**) Bar plots of major eukaryotes by group. (**E**) Distribution of each bacterial phyla by CDI status. (**F**) Distribution of each bacterial phyla by group. (**G**) Distribution of eukaryotes by CDI status. (**H**) Distribution of eukaryotes by group. Figure created on R studio with ggplot package^64,69^.

During the taxonomic assignment, a total of 75,126 amplicon sequencing variants (ASVs) were found for the 16S-rRNA marker, corresponding to 49 phyla and 659 genera (74,594 ASVs (99.29%) for bacteria and 532 ASVs (0.71%) corresponding to Archaea); while 11,265 ASVs were found for the 18S-rRNA marker, corresponding to 54 classes and 623 genera (3396 ASVs (30.14%) for Fungi). Initially, samples were analyzed based on their CDI status (positive/negative). Our analysis showed 90% of bacteria in the samples belonged to the phyla Bacteroidetes, Firmicutes, or Proteobacteria, while approximately 9% belonged to the phyla Acidobacteria, Actinobacteria, Fusobacteria, Spirochaetes, or Verrucomicrobia (Fig. 1A). In general, the bacterial community composition of the CDI-negative group showed a predominance of Firmicutes and Bacteroidetes, while the pattern of CDI-positive patients was characterized by a decrease of Firmicutes and a small increase in the relative abundance of Proteobacteria and Verrucomicrobia compared to the CDI-negative group (Fig. 1A,E). With the eukaryotes, similar patterns were seen in both groups, with a predominance of organisms belonging to the Fungi and Metazoa kingdoms. However, we observed a significant increase in the relative abundance of microorganisms belonging to the phylum Opalozoa in the CDI-positive patients (*p* = 0.04613) (Fig. 1B,G).

The analysis of the composition of the intestinal bacteria within the four study groups (HCFO/+, CO/+, HCFO/−, and CO/−) showed a decrease in the relative abundance of Bacteroidetes in the HCFO/− group with respect to the CO/− group. Similarly, there was a significant decrease in Firmicutes in the HCFO/+ group with respect to the CO/+ and CO/− groups (*p* values; CO/− vs HCFO/+  = 0.00773, CO/+ vs HCFO/+  = 0.028) (Fig. 1C,F). The distribution of the Archaea genera within the study groups was deepened, showing more than 10 different genera, among which *Methanobrevibacter* and *Methanosaeta* stood out as the most abundant (Figure S2). The HCFO/+ group was characterized by a higher abundance of most archaea, except *Methanobrevibacter*, as the HCFO/− group presented a marked increase in the relative abundance of this genus with respect to the other groups evaluated. Finally, the compositions of the eukaryotes were more uniform throughout the evaluated groups, with no differences between the organisms belonging to the observed classes (Fig. 1D,H).

### Alpha and beta diversity with no differences between groups

When we analyzed the diversity indices for both bacteria and eukaryotes among patients with positive and negative CDI results, we observed Shannon and Simpson indices indicative of low diversity, with no significant differences between patients (Figure S3A and S3B). A similar pattern was evidenced when we analyzed the diversity indices among the four study groups: no significant differences were found among the groups, which all had relatively low diversities (Figure S3C and S3D). Finally, in the case of beta diversity, we found no characteristic patterns that would allow for clear spatial groupings between the members of the different groups (Figure S3E and S3F).

### Robust differences between the genera commonly found in the gut

**Figure 2:**
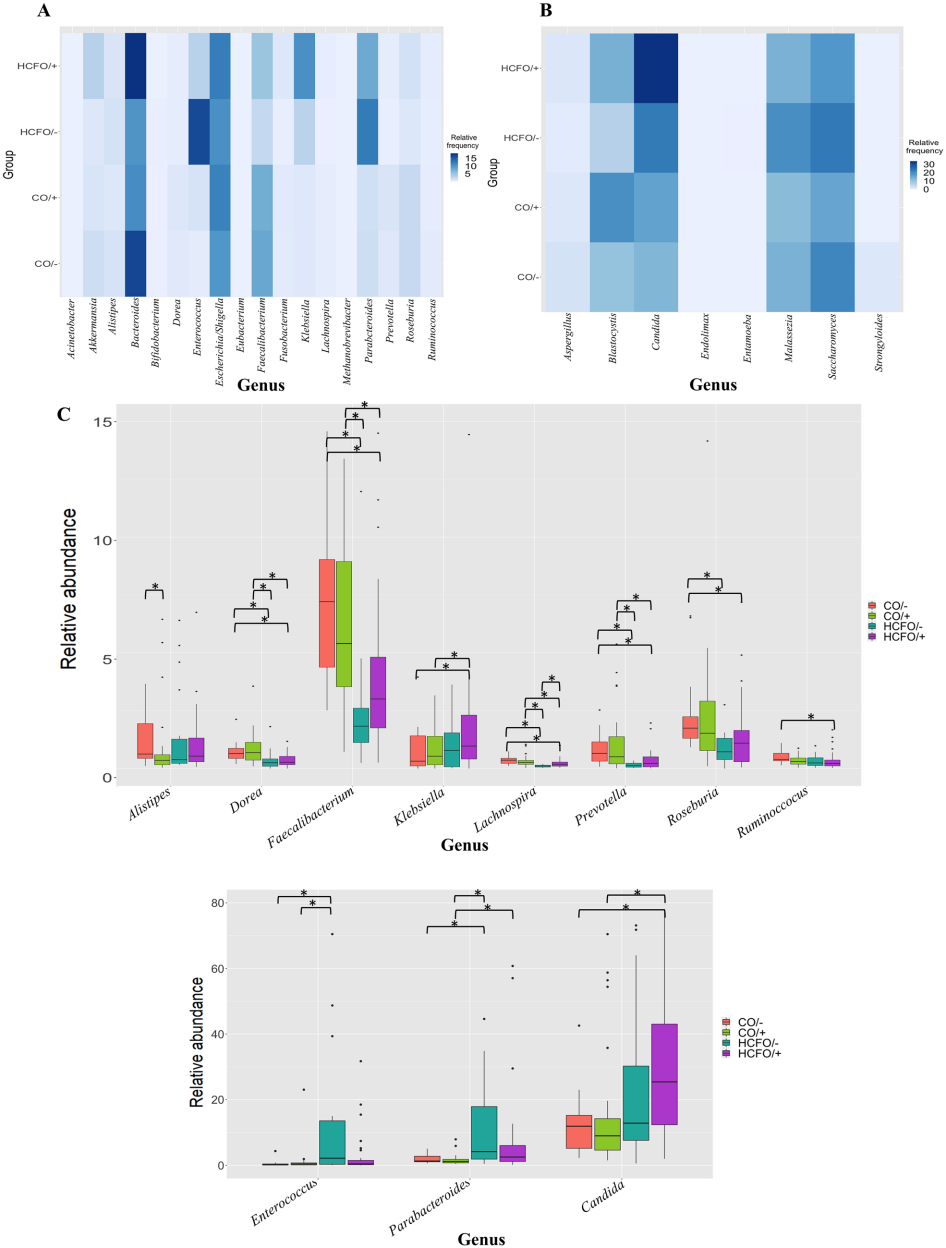
Changes in bacteria and eukaryotes commonly found in the gut microbiota. (**A**) Heatmap of bacterial genera by group. (**B**) Heatmap of eukaryotic genera by group. (**C**) Boxplot showing the differences between groups by relative abundances of each genus. Statistical differences (Kruskall–Wallis test; Post-hoc: Dunn test with Benjamini–Hochberg correction and a confidence level of 95%) (*p* < 0.05) are indicated by an asterisk mark (*). Figure created on R studio with ggplot package^64,69^.

In the present research, 18 of the bacterial genera and eight of the most common eukaryotic genera from the human intestinal tract^19,20^ were compared among the groups. Based on these genera, heatmaps were constructed, in which differences in the relative abundances were investigated (Fig. 2A,B). From the evaluation of the genera, significant differences (*p* < 0.05) in abundances were revealed for 10 bacterial genera, including *Dorea*, *Faecalibacterium*, *Lachnospira*, and *Prevotella*, and one eukaryotic genus, *Candida* (Fig. 2C). The remaining eight bacterial and seven eukaryotic genera did not show significant differences in abundance between the studied groups (Figure S4). Despite this, some eukaryote genera displayed striking patterns. Among these, the increase in the relative abundance of *Blastocystis* in the CO/+ and HCFO/+ groups with respect to the CDI-negative groups was prominent. Furthermore, the HCFO/− group showed an increase in the relative abundance of *Saccharomyces* and *Malassezia* genera with respect to the other groups (Figure S2).

When we analyzed the differential distribution of *Candida, Saccharomyces*, and *Malassezia* spp.; different configurations were observed for each genus in each of the study groups. In the case of *Candida*, an increase was observed in the HCFO/− and HCFO/+ groups, where more than 30 different species were associated to several ASVs (> 99% similarity), with *C. albicans* and *C. glabrata* as the most abundant (Figure S2B). In the case of *Saccharomyces*, only the presence of ASVs corresponding to *S. cerevisiae* was evidenced as having a comparable distribution among the different groups (Figure S2C). Finally, ASVs relating to nine *Malassezia* species were identified, with the most abundant ASVs corresponding to *M. restricta*, followed by *M. globosa* and *M. furfur*, which had obvious increased abundances in the intrahospital-acquired diarrhea groups (Figure S2D).

### Correlogram between bacteria and eukaryotes

**Figure 3:**
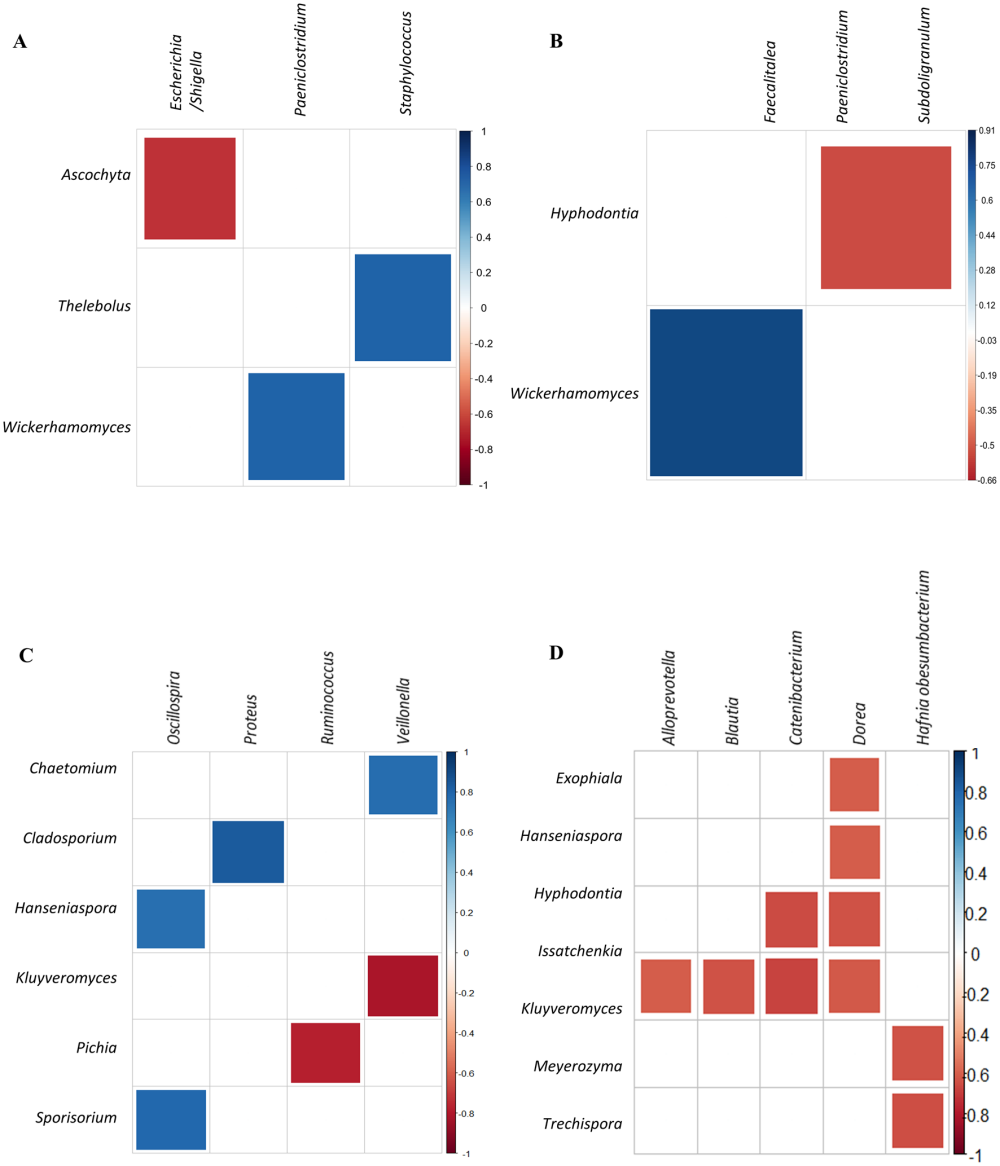
Possible interactions between kingdoms. Correlogram plots between bacteria and eukaryotes. ASVs corresponding to the most abundant phyla (Bacteroidetes, Firmicutes, Proteobacteria, Ascomycota and Basidiomycota) were compared. Outliers were deleted to only compare the ASVs corresponding to the most abundant genera (Spearman’s rho correlation method with Benjamini–Hochberg correction). Only were considered strong correlations (− 0.7 < ρ > 0.7; *p* value < 0.05). (**A**) CO/− group. (**B**) CO/+ group. (**C**) HCFO/−group. (**D**) HCFO/+ group. Figure created on R studio with psych package^64,72^.

To identify the possible interactions occurring between the intestinal bacteria and eukaryote genera, correlograms were generated using the reads from each of these microorganism groups. In general, an inverse correlation was observed in all study groups between some genera of fungi and bacteria of the phylum Firmicutes (Fig. 3). Strikingly, the CDI-negative groups presented the highest number of significant correlations (Figs. 3A,C). Similarly, in the HCFO/+ group, there were many inverse correlations between bacteria of the genus *Dorea* and various fungal genera (Fig. 3D).

### Identification of potential biomarkers

**Figure 4:**
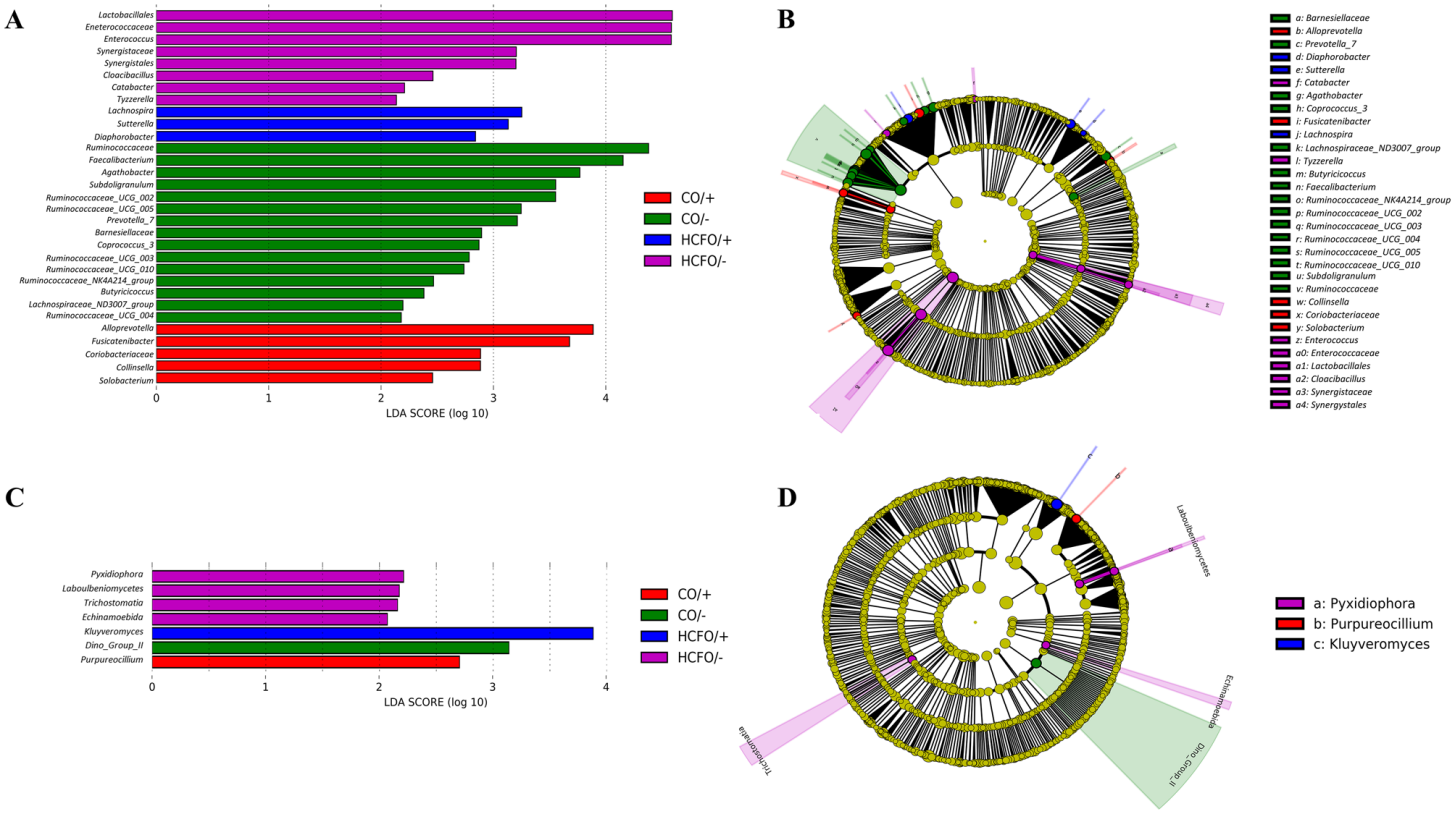
Effect of size measurements (LEfSe) at phylum and genus levels. Linear discriminant analysis (LDA) combined with LEfSe showing a list of possible biomarkers that enable discrimination between groups. LDA ≥ 2.0 and *p* < 0.05 were considered significant in Kruskal–Wallis and pairwise Wilcoxon tests. (**A**) Distinct bacterial phyla and genera biomarkers by group. (**B**) Cladogram reporting the bacterial taxa (highlighted by small circles and shading) showing different abundance values in the groups. Circles’ diameters are proportional to the taxon’s abundance, and the shadow size is proportional to the effect size. (**C**) Distinct eukaryotic phyla and genera biomarkers by group. (**D**) Cladogram reporting eukaryotic taxa showing different abundance values in the groups. Figure created on Galaxy platform^73,74^.

Finally, LEfSe analyzes were performed to determine the characteristic microbiological profiles of the four groups by considering the most abundant taxa in each of the study populations to be potential biomarkers (linear discriminant analysis [LDA] score > 4, *p* < 0.05, non-strict analysis). In the CO/− group, a differential predominance of bacteria belonging to three families of the phylum Firmicutes was found compared with the other groups: Ruminococcaceae, Lachnospiraceae, and Clostridiaceae had LDA scores > 4, with the Ruminococcaceae being the most characteristic within this group, followed by bacteria belonging to the genus *Faecalibacterium*. However, the patients of the CO/+ group showed a greater abundance of bacteria belonging to the genera *Alloprevotella* and *Fusicatenibacter*, which showed an LDA score > 3, and represented the greatest difference in this group compared with other groups (Fig. 4A,B).

In the HCFO/− group, the most abundant bacteria belonged to the order Lactobacillales, and the most important representative within this order was the genus *Enterococcus*, with an LDA score > 4 for both the order and genus. Finally, the HCFO/+ group was characterized by a greater abundance of *Lachnospira* bacteria; however, the lowest LDA scores and the least number of differentiating characteristic groups were found in this group (Fig. 4A,B). Finally, Archaea were not evaluated due to the low abundance.

In the case of eukaryotes, the number of biomarkers per group was considerably reduced. The CO/− group was characterized by an abundance of protists belonging to Dino Group II; while, for the CO/+ group, we evidenced an increase in fungi of the genus *Purpureocillium* with a moderate effect size. The HCFO/− group showed the smallest effect sizes in all its biomarkers, which were the most diverse, as they ranged from fungi such as *Pyxidiophora* to protists belonging to the Trichostomatia class. The HCFO/+ group was found to have the largest effect size with its unique biomarker, *Kluyveromyces* (Fig. 4C,D).

## Discussion

In terms of the composition of the microbiota, an increase in the relative abundance of bacteria belonging to the phyla Bacteroidetes and Firmicutes was observed in CDI-positive patients with respect to CDI-negative patients (Fig. 1). This increase is contrary to what was previously discovered in various studies that found intestinal microbiota profiles were characterized by a decrease in these phyla^21–24^. This decrease is supported by the roles that these groups of microorganisms play in carbohydrate metabolism and the production of short-chain fatty acids (SCFAs), as well as their contribution to the regulation of the immune system, which hinders the development of vegetative forms of *C. difficile*. Therefore, a decrease in Bacteroidetes and Firmicutes facilitates the development of *C. difficile* infections^25,26^. This discrepancy in the results may be explained by the fact that a greater increase in the relative abundance of these phyla, especially Firmicutes, was seen in patients with community-acquired diarrhea (CO/+) (Fig. 1). This may account for the smaller impact on the intestinal microbiota of the circulating strains in this population, and the physical and nutritional conditions of patients with community-acquired diarrhea may provide a more stable intestinal microbiota composition and, therefore, less severe forms of CDI. However, since there are no clinical data associated with the patients, further research is needed to reveal the true impact of the source of the *C. difficile* infection and associated clinical factors.

Another important result in the composition of the microbiome was the increased abundance of Proteobacteria in patients with CDI compared with uninfected patients (Fig. 1). The increase these bacteria has been previously reported for CDI, suggesting that these microorganisms take advantage of the imbalance of the microbiota to proliferate and may even cause the exacerbation of symptoms^9,23,24^. In this sense, it is necessary to evaluate in future studies the abundance of these phyla in a non-diarrheal control group, which was not the objective of our analysis. This is also corroborated with the diversity of both the bacterial and eukaryotic microbiota that was low in all groups studied (Figure S3). This can be related with diarrhea presentation.

In the case of eukaryotes, the predominance of fungi in the eukaryotic microbiome composition (Fig. 1) agrees with previous descriptions, as these organisms are the main component of the eukaryotic intestinal microbiota^27–29^. However, the absence of differences between the studied groups contrasts with previous reports in which an increased ratio between Basidiomycota/Ascomycota was associated with the pathogenesis of colorectal cancer and inflammatory bowel disease^30^. The absence of these changes could be due to the presence of diarrhea that may help to hide differences between the groups. This highlights the need to compare the findings with those from patients without diarrhea in future studies.

As previously discussed, the health status of patients who develop intrahospital diarrhea is usually marked by multiple treatments and interventions that can facilitate the emergence of various pathogens, such as *C. difficile*^9,23,31^. However, the absence of this pathogen in some individuals of the HCFO/− group suggests that alternative mechanisms may contribute to the homeostasis of the intestinal microbiota, which should be investigated in a future study. One of the proposed mechanisms is related to the marked increase in *Methanobrevibacter* in this group (Figure S2), involved in production of SCFAs from carbohydrates^32–34^, some of which act as growth inhibitors of *C. difficile*^17^. This suggests that some archaea could contribute to supply the function of some bacterial families that produce these components and were found to be diminished in this group. Therefore, there may be a relationship between the increase in *Methanobrevibacter* (Figure S2) and protection against CDI, the mechanism of which should be addressed in future research. In addition, studies should consider other methanogenic Archaea, which contrastingly increased in the HCFO/+ group, denoting a possible genus-specific role not described so far.

Diarrhea, associated with an imbalance of microbiota, may contribute to the increase in certain opportunistic microorganisms. In this regard, *Candida* and *Malassezia* genera in the HCFO/+ and HCFO/− groups (Figures S2 and S4) could have taken advantage of the disruption of the microbiota to consolidate their populations, although these tend to be pathobionts and are innate components of the intestinal microbiota^30,35,36^. For example, more than 30 species of *Candida* were identified (Fig. 2 and Figure S2), prominently *C. albicans* and *C. glabrata*; the latter are recognized for their ability to alter the microbiota of immunocompromised individuals^37,38^, and their growth is favored by the proliferation of aerobic bacteria, especially of the Enterobacteriaceae family^39^, which were increased in the HCFO groups (Fig. 2). In a similar way, the *Malassezia* species, among which *M. restricta* was prominent (Figure S2), are known for their ability to exacerbate the severity of colitis in Crohn’s disease without causing alterations to the microbiota^40^. This indicates a complex relationship between kingdoms in which the microbiota is altered by some bacteria and eukaryote groups to facilitate their proliferation while utilizing the energy sources available as a result of the decrease in other beneficial groups. Additionally, the modulation of immunity at the intestinal level by some bacteria could be used by opportunistic pathogens to proliferate, leading to a state of intestinal ecosystem imbalance, as occurs in other inflammatory diseases^38,41–43^. Similar phenomena can occur as a result of eukaryotes that facilitate infection by pathogenic bacteria^44^.

Contrary to the findings described above, the presence of some eukaryotes, such as *Blastocystis*, has been associated with an increase in the diversity of the bacterial microbiota and of beneficial bacteria groups such as *Faecalibacterium* and *Roseburia*^45–47^. This positive modulation of the intestinal microbiota is related to the development of an anaerobic environment, which is necessary for the growth and development of this protozoan and is generated by beneficial bacteria through the production of SCFAs such as butyrate. The SCFAs are consumed by the colonocytes, increasing oxidative phosphorylation and, thus, decreasing the amount of oxygen available in the intestinal lumen^48^. This was exemplified by the CO/+ group, in which there was an increase in the relative abundance of *Blastocystis* (Figure S4) accompanied by an increase in beneficial bacteria such as *Faecalibacterium*, *Lachnospira*, *Prevotella*, and *Roseburia* (Fig. 2). The above suggests that the co-occurrence of *Blastocystis* and *C. difficile* attenuates the negative impact of CDI on the intestinal microbiota. The effects of this attenuation on clinical manifestations should be studied in depth in future research.

The co-relationships between bacteria and eukaryotes are marked by multiple interactions, both synergistic and antagonistic, and competition for energy sources^30,36,38^. The correlograms in this study represent the complex relationships between the various bacterial and eukaryotic genera (Fig. 3). These relationships in the CDI-negative groups may denote closer interactions, as occurs in ulcerative colitis^38,42^. Furthermore, the inverse correlations observed in the CDI-positive groups reinforce the previous hypothesis that some microorganisms proliferate by exploiting the energy sources available from the depletion of other microorganisms and suggest a greater antagonism between the different kingdoms, as occurs in Crohn’s disease^49^. Imbalances in the microbiota similar to those shown here, which are usually associated with the deterioration of the immune system and metabolic homeostasis in critically ill patients at risk of developing sepsis^50^, are generated mainly by the presence of patients at ICUs, which as mentioned above, can profoundly alter the delicate balance of the intestinal ecosystem. Moreover, polymicrobial interactions that occur within biofilms have been associated with the progression of some diseases, such as colorectal cancer, prostatitis, and cystic fibrosis^51–53^, highlighting once again the importance of further studying their potential roles.

To differentiate between the studied groups based on the abundance profiles of gut microbiota members, prokaryote and eukaryote biomarkers were established (Fig. 4). As has been shown throughout the study, each group showed characteristic profiles that could account for the degree of homeostasis of the intestinal microbiota. An example of this occurred in the HCFO/− group, in which the increase in bacteria belonging to the Lactobacilalles, Enterococcaceae, and Enterococcus groups showed disruption of the microbiota characteristic of diarrhea associated with irritable bowel syndrome^22^. Whereas the increase in bacteria belonging to the Ruminococcaceae family, proposed as biomarkers for the CO/− group, suggest a greater degree of balance within the intestinal microbiota driven by the production of SCFAs, such as butyrate^10^. A similar profile occurred in the other CDI-negative group, in which the presence of potentially beneficial bacteria was associated with the presentation of less severe symptoms and better resolution of the disease^54,55^. Finally, the increase in *Alloprevotella* noted in the HCFO/+ group agrees with findings from previous studies on other inflammatory bowel diseases, in which an increase in Prevotellaceae family members was associated with the development of inflammation and colitis, and *Alloprevotella* was suggested as a biomarker for the identification of these pathologies.

We found that most of the eukaryotic biomarkers corresponded to fungi not usually found at the intestinal level^56^, which suggests that the number and diversity of eukaryotic sequences available in the databases should be increased to allow for more accurate taxonomic assignment based on the 18S-rRNA marker and thus achieve greater precision in the description of this community^55^. Despite the above, the suitability of *Kluyveromyces* as a biomarker of HCFO/+ group suggests immunosuppression is a key factor in the development of this type of opportunistic infection^57,58^, as eukaryotic infections are associated with both the multiple clinical and therapeutic interventions to which patients may be exposed at the hospital, generating a general alteration of their health status.

The present investigation aimed to evaluate the roles of other microorganisms, such as eukaryotes, with unclear effects in CDI based on patients with diarrhea. Despite the absence of clinical and sociodemographic data, there were some characteristic differential profiles among the four evaluated groups that deserve to be studied in greater depth to reveal the roles of other microorganisms, such as viruses, and identify potentially relevant virulence and resistance markers. In the future, it is necessary to conduct additional analyses including a cohort of control patients (without diarrhea) to have a most complete view about the changes in gut microbiota composition under different scenarios. Additionally, the use of techniques such as metagenomic sequencing and interactome analysis will be required to complement the data presented here and to provide a more holistic understanding of CDI and the role of the intestinal microbiome in its establishment, development, and recovery.

## Methods

### Ethical considerations

The current project was conducted with the approval of the Research Ethics Committee of the Universidad del Rosario (Approval Act No. 339). This study was considered low risk according to Resolution 8430 of 1993 of the Ministry of Health of Colombia. The samples were coded to protect the identity of the patients in accordance with national ethical guidelines and the Declaration of Helsinki. The duration of diarrheal symptoms was the only data obtained from the clinical history of the patients and was directly associated with the coding of the sample. Informed consent was obtained for the use of the sample in research in accordance with what was authorized by the ethics committee. Data concerning age and sex were collected only from the HCFO groups.

### Study population

A total of 98 DNA samples were selected from the biobank of the Microbiological Research Group—UR (GIMUR). These stool samples were obtained in the framework of the “*Clostridioides difficile* characterization in Colombia” project from patients with diarrhea, the main symptom of *C. difficile* infection (CDI). Each sample was assigned to a group according with the location of diarrhea acquisition, CO- or HCFO-acquired, according to the protocols of the Society for Healthcare Epidemiology of America and the Infectious Diseases Society of America^31^.

Sample collection and transportation procedure is detailed at supplementary information section. DNA extraction protocol and CDI status identification were obtained from Muñoz et al.^59^. Briefly, DNA extraction was performed using the Stool DNA Isolation Kit (Norgen, Biotek Corporation, Thorold, Canada) following the manufacturer’s instructions. Conventional PCR was performed for the detection of CDI using the markers 16S-RNA and glutamate dehydrogenase (GDH), as reported elsewhere^59^. The results were visualized by 2% agarose gel electrophoresis. Based on these results, the following groups were stablished:

- Group 1: CDI-positive samples from HCFO (n: 30).
- Group 2: CDI-positive samples from CO (n: 30).
- Group 3: CDI-negative samples from HCFO (n: 18).
- Group 4: CDI-negative samples from CO (n: 20).

### DNA quality control and sequencing process

The extracted DNA was subjected to quality control by 2% agarose gel electrophoresis to verify the integrity of the DNA. Additionally, the concentration was evaluated using a NanoDrop/2000/2000c spectrophotometer (Thermo Fisher Scientific, Massachusetts, USA). A 260/280 relationship between 1.8 and 2.0 and a minimum concentration of 20 ng/µL was verified.

Paired-end sequencing was performed on the Illumina HiSeq platform (PE 250 Platform) with a depth of 100.000X at the facilities of Novogene Corporation Inc. (Shanghai, China) using primers targeting the hypervariable V4 region of the 16S-rRNA marker specific for bacteria and Archaea 515-F (5′-GTGCCAGCMGCCGCGGTAA-3′) and 806-R (5′-GGACTACHVGGGTWTCTAAT-3′)^60^. For the description of eukaryotic communities, we used primers targeting the hypervariable region of 18S-rRNA 528F (5′-GCGGTAATTCCAGCTCCAA-3′) and 706R (5′-AATCCRAGAATTTCACCTCT-3′)^61^.

### Taxonomic assignment

Initially, a quality control step was performed to determine the quality of the reads from the sequencing process. The average number of reads per sample, phred score, frequency of unassigned bases, and content of adapters in the samples were analyzed. Subsequently, the barcodes and primers were removed using the QIIME2 tool^62,63^ before proceeding with taxonomic assignment using the DADA2 tool in R studio^64^, following the default pipeline^65^. The assignment was performed by comparing the sequences obtained for the 16S-rRNA marker against the 16S-rRNA SILVA version 132 database^66^, and the sequences obtained for the 18S-rRNA marker against the Protist Ribosomal Reference database (PR2)^67^. Finally, rarefaction curves were performed to determine the sufficiency of the sequencing depth to ascertain the microbial diversity of the samples using the ranacapa package of the R Studio program^68^.

### Diversity analysis

Based on the ASVs resulting from the taxonomic assignment, we created relative abundance graphs (bar plots) of the different phyla and genera in both the CDI-positive and -negative patients. Subsequently, alpha (Shannon and Simpson) and beta (NMDS from Bray–Curtis similarity index matrices) diversity analyzes were performed using R studio’s phyloseq package and were later graphed using ggplot2^69^ and reshape2^70^.

### Heatmaps, correlations, and biomarker search

A heatmap was generated with the most relevant bacterial and eukaryotic genera at the intestinal level^19,20^ to determine the relative abundances in each group. From the findings from the heatmaps, boxplots were created to determine the differences between the studied groups with respect to the genera investigated. The differences between the groups in terms of abundance of archaea and some genera of eukaryotes was determined by creating chord diagrams using the circlize package^71^ in R studio. Statistically significant differences between the studied groups were evaluated using the Kruskal–Wallis test with respective post-hoc analyses with the Dunn test using Benjamini–Hochberg correction with a confidence level of 95%. Likewise, correlogram graphs were made between ASVs corresponding to the most abundant phyla (Bacteroidetes, Firmicutes, Proteobacteria, Ascomycota and Basidiomycota). Based on these, a filter of sub-represented data was carried out, eliminating all those ASVs corresponding to genera whose sum of reads was less than 1,000, as well as those ASVs corresponding to genera whose reads were not present in at least 25% of the samples. These filters were carried out to reduce potential technical bias and to ensuring that comparisons were made between ASVs that were present in the groups and not in single samples. The correlation matrix was constructed using the psych package on R software^72^ applying the spearman method with Benjamini–Hochberg correction. We considered only strong correlation values greater than 0.7 and less that − 0.7 (Spearman Rho strong correlation) and select statistically significant (*p* < 0.05) at the moment of establishing a correlation between the ASVs evaluated. Finally, we performed multiple comparisons among the different taxa of the groups to identify potential biomarkers using an LDA of effect of size (LEfSe), which was performed on the Galaxy platform^73,74^, following the indications of the framework.

## Supplementary Information

Supplementary Information.

## Data Availability

The 16S-rRNA and 18S-rRNA gene sequencing data used in this study are available through the National Center for Biotechnology Information (NCBI) Sequence Read Archive: https://ncbi.nlm.nih.gov/sra under accession number PRJNA679727.
