# Supplementary material for: Gut microbiota composition in health-care facility-and community-onset diarrheic patients with Clostridioides difficile infection
Source: Sci Rep. 2021 May 25;11:10849. doi: 10.1038/s41598-021-90380-7 (PMC8149855; doi:10.1038/s41598-021-90380-7)
Supplement: Supplementary file 1 — Supplementary Information. [file 41598_2021_90380_MOESM1_ESM.pdf]

Gut microbiota composition in health-care facility-and community-onset diarrheic patients with *Clostridioides difficile* infection

Giovanny Herrera[1], Laura Vega[1], Manuel Alfonso Patarroyo[2,3,4], Juan David Ramírez[1], Marina Muñoz[1*]

[1] Grupo de Investigaciones Microbiológicas – UR (GIMUR), Departamento de Biología, Facultad de Ciencias Naturales, Universidad del Rosario, Bogotá, Colombia.

[2] Molecular Biology and Immunology Department, Fundación Instituto de Inmunología de Colombia (FIDIC), Bogotá, Colombia

[3] Microbiology Department, Faculty of Medicine, Universidad Nacional de Colombia, Bogotá D.C. 111321, Colombia

[4] Health Sciences Division, Main Campus, Universidad Santo Tomás, Bogotá D.C. 110231, Colombia

*Corresponding author: claudia.munoz@urosario.edu.co

Supplementary figures

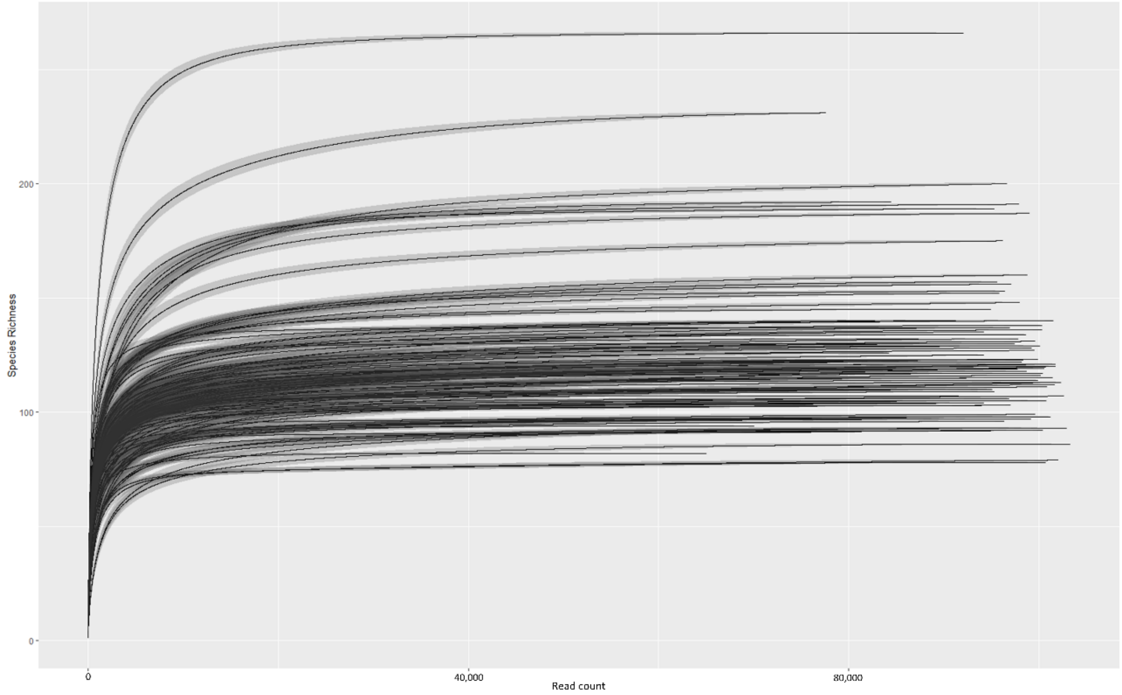

Supplementary figure 1. Rarefaction curve showing the species richness in function of depth read by sample. Figure created on R studio with ranacapa package [64,68].

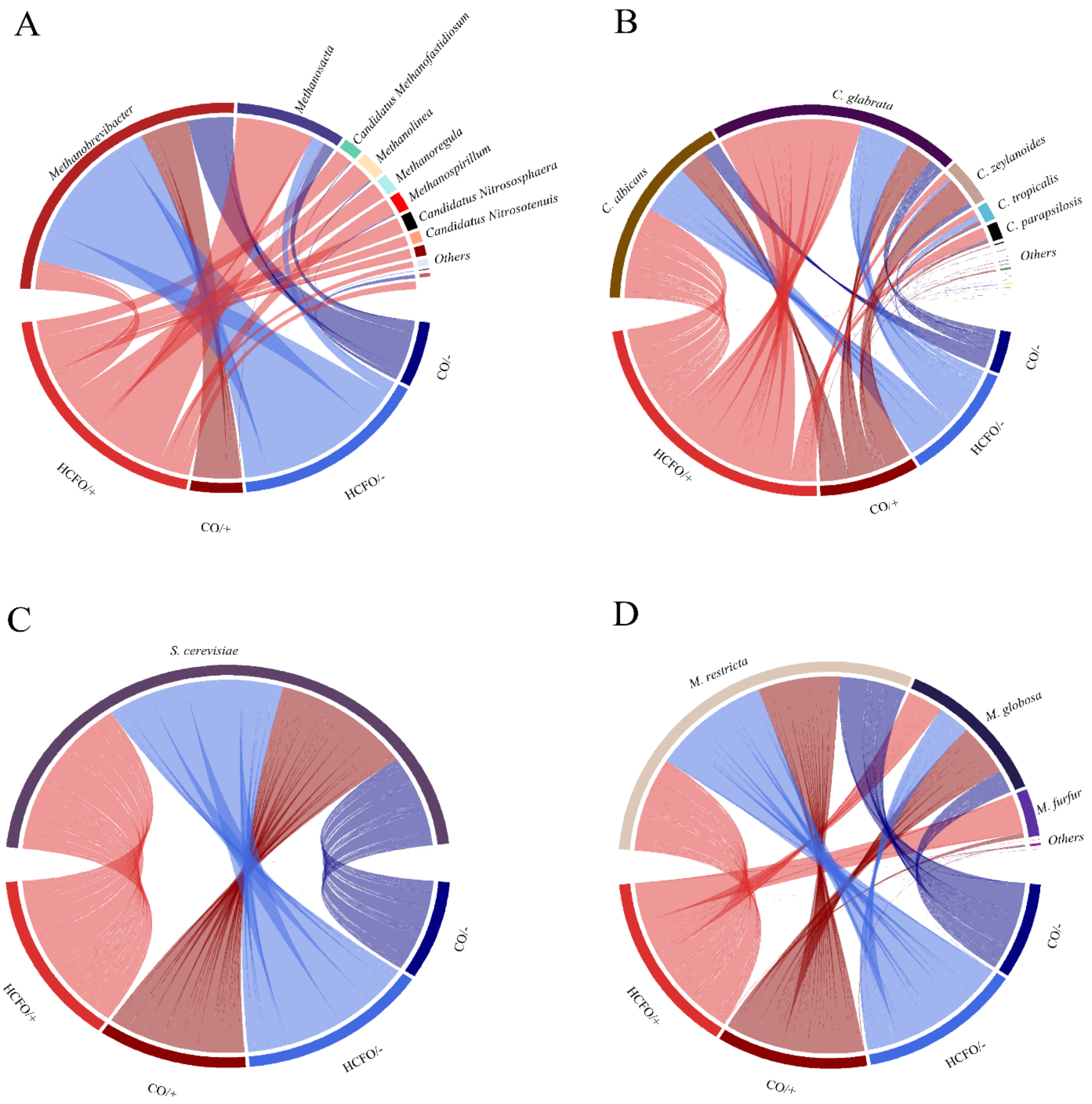

Supplementary figure 2. Circos plot showing differential abundance of Archaea genera and eukaryotic species by group. A. Archaea genera by group. B. *Candida* species by group. C. *Saccharomyces* species by group. D. *Malassezia* species by group. CO: community-onset; HCFO: Healthcare facility-onset; +: positive result for *Clostridiodes diffi*cile infection (CDI); -: negative result for CDI. Figure created on R studio with circlize package [64,71].

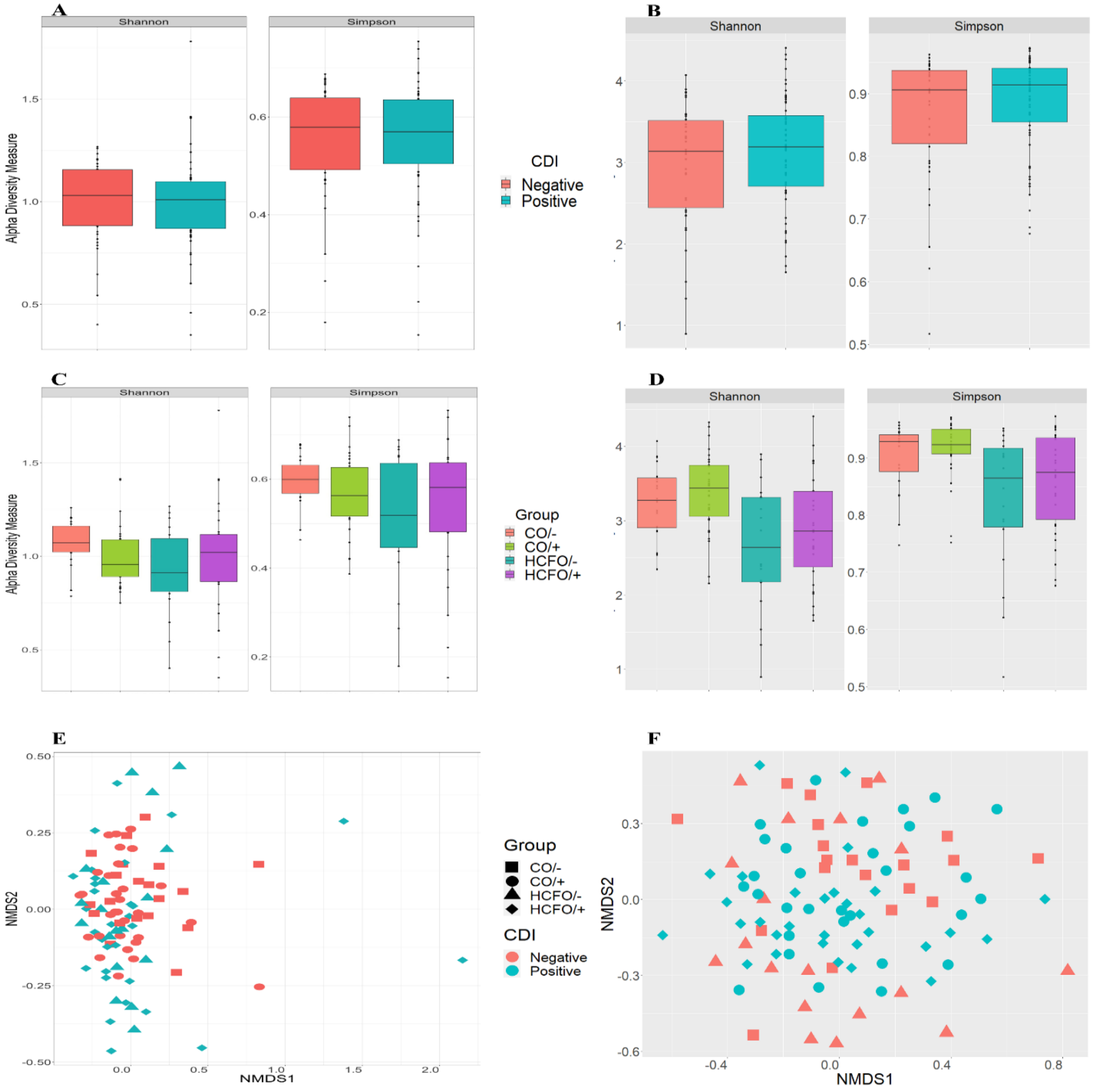

Supplementary figure 3. No differences between groups in alpha and Beta diversity. A. Boxplot of Shannon and Simpson's diversity indexes by CDI status for bacteria. B. Boxplot of diversity indexes by group for bacteria. Statistical differences were evaluated with Kruskall-Wallis test; Post-hoc: Dunn test with Benjamini-Hochberg correction and a confidence level of 95% ($p < 0.05$). C. NMDS plot of samples separated by group and CDI status for bacteria. D. Boxplot of diversity indexes by CDI status for eukaryotes. B. Boxplot of diversity indexes by group for eukaryotes. C. NMDS plot of samples separated by group and CDI status for eukaryotes. CO: community-onset; HCFO: Healthcare facility-onset; +: positive result for *Clostridiodes difficile* infection (CDI); -: negative result for CDI. Figure created on R studio with ggplot and reshape packages [64,69,70].

A

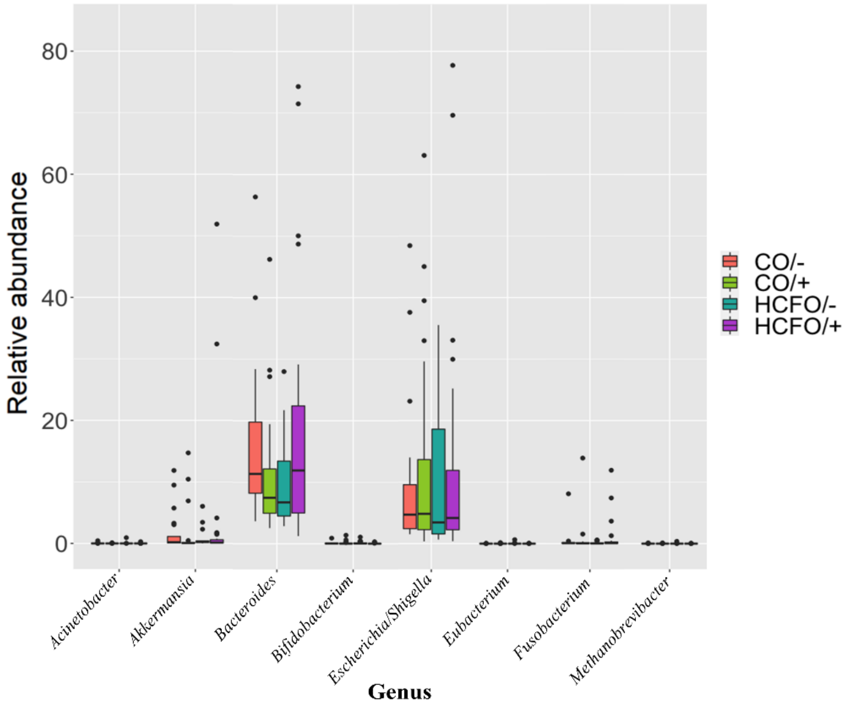

B

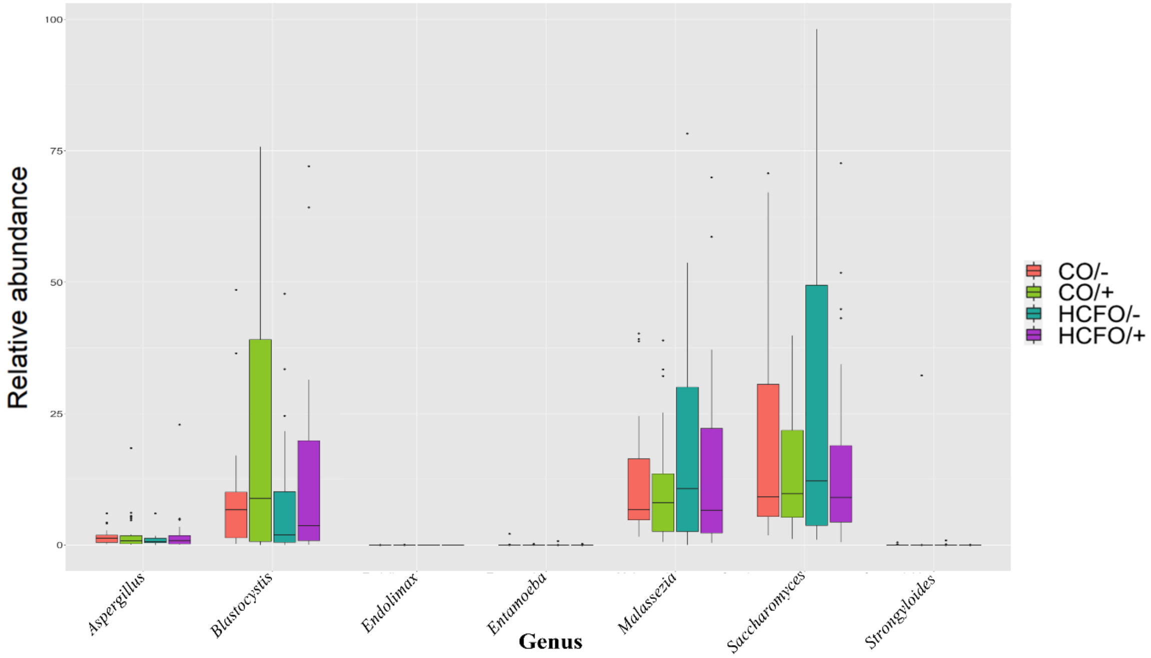

Supplementary figure 4. Boxplot showing no differences between groups in different bacterial and eukaryotic genera by group. Statistical differences were evaluated with Kruskall-Wallis test; Post-hoc: Dunn test with Benjamini-Hochberg correction and a confidence level of 95% ($p < 0.05$). CO: community-onset; HCFO: Healthcare facility-onset; +: positive result for *Clostridiodes diffi*cile infection (CDI); -: negative result for CDI. Figure created on R studio with ggplot package [64,69].

Supplementary text 1. Sample collection and transportation procedures

Samples analyzed in this study were obtained from patients with diarrhea (the main symptom associated with *Clostridiodes difficile* infection), who signed an informed consent to participate in the study after knowing the objective of the study and its implications. Individuals from the community (CO) were required to collect their own stool sample; nurse practitioners from the intensive care units collected samples from HCFO individuals. All diarrheic feces samples were collected in sterile recipients (without transport media), refrigerated (2-8ºC) and stored until being processed within the first 72 hours following collection. Each sample was transported in a hermetic recipient conserving the cold chain to the Universidad del Rosario's Microbiology Laboratory where they were homogenized by mechanical disruption using sterile scrapers, as the initial processing step. All the samples that were not processed immediately, were stored at -20°C.
